# From Distress Screening to Uptake: An Italian Multicenter Study of Cancer Patients

**DOI:** 10.3390/cancers13153761

**Published:** 2021-07-27

**Authors:** Elena Meggiolaro, Silvia De Padova, Federica Ruffilli, Tatiana Bertelli, Marina Bragagni, Sabrina Prati, Lidia Pisotti, Ilaria Massa, Flavia Foca, Stefano Tamberi, Ugo De Giorgi, Luigi Zerbinati, Elisa Tiberto, Luigi Grassi

**Affiliations:** 1 Psycho-Oncology Unit, IRCCS Istituto Romagnolo per lo Studio dei Tumori (IRST) “Dino Amadori”, 47014 Meldola, Italy; silvia.depadova@irst.emr.it (S.D.P.); federica.ruffilli@irst.emr.it (F.R.); tatiana.bertelli@irst.emr.it (T.B.); 2 Nursing and Technical Administration, IRCCS Istituto Romagnolo per lo Studio dei Tumori (IRST) “Dino Amadori”, 47014 Meldola, Italy; marina.bragagni@irst.emr.it (M.B.); sabrina.prati@irst.emr.it (S.P.); lidia.pisotti@irst.emr.it (L.P.); 3 Healthcare Administration, IRCCS Istituto Romagnolo per lo Studio dei Tumori (IRST) “Dino Amadori”, 47014 Meldola, Italy; ilaria.massa@irst.emr.it; 4 Unit of Biostatistics and Clinical Trials, IRCCS Istituto Romagnolo per lo Studio dei Tumori (IRST) “Dino Amadori”, 47014 Meldola, Italy; flavia.foca@irst.emr.it; 5 Medical Oncology Unit, Ospedale degli Infermi, 48018 Faenza, Italy; stefano.tamberi@auslromagna.it; 6 Department of Medical Oncology, IRCCS Istituto Romagnolo per lo Studio dei Tumori (IRST) “Dino Amadori”, 47014 Meldola, Italy; ugo.degiorgi@irst.emr.it; 7 Department of Neuroscience and Rehabilitation, Institute of Psychiatry, University of Ferrara, 44121 Ferrara, Italy; zrblgu@unife.it (L.Z.); elisa.tiberto.pd@gmail.com (E.T.); luigi.grassi@unife.it (L.G.)

**Keywords:** distress screening, cancer patients, psychosocial uptake, referral, screening policy

## Abstract

**Simple Summary:**

An evaluation process and adequate referrals are an important part of a distress screening program but insufficient consideration is given to referrals and uptake of available supportive services. Identifying the reasons for accepting or refusing help is needed to implement a screening-for-distress policy in a clinical cancer setting, as confirmed in the present study. It is vital to reach and motivate the highest possible number of patients to be referred to psycho-oncology services when needed. A multidisciplinary approach could help to raise awareness of the benefit of screening for distress, the implementation of which would improve uptake.

**Abstract:**

Introduction: Little consideration is given to the referral and uptake of available supportive services after distress screening. However, identifying the reasons for accepting or refusing help is mandatory for implementing a screening policy. The present study explored the practical usefulness of and potential barriers to the application of distress management. Methods: 406 cancer patients were consecutively selected and asked to complete the Distress Thermometer (DT) and Problem Check List (PL). All patients with a DT score ≥6 were invited for a post-DT telephone interview with a trained psychologist. Results: The 112 patients who refused to take part were more often older, retired, at a more advanced stage of illness, and with no previous experience of psychological intervention with respect to those who accepted. Of the 78 patients with a score ≥6 who were referred to the Psycho-Oncology Service, 65.4% accepted the telephone interview. Twenty-two patients rejected the initial invitation immediately for various reasons including logistic difficulties, physical problems, and feeling embarrassed about opening up to a psychologist. Conclusions: Our study confirms that screening *per sé* is insufficient to deal with the problem of distress and that more emphasis should be placed on implementing referral and treatment.

## 1. Introduction

Cancer diagnosis and treatment often lead to patients experiencing considerable emotional consequences and psychological problems. Literature data have highlighted that patients undergo profound changes in both psychosocial and physical states due to diagnosis, communication and treatments [1,2]. There is strong evidence that distress can negatively impact quality of life and is associated with poorer clinical outcome in cancer patients [3,4]. In fact, significant levels of untreated distress are associated with limited adherence to treatment recommendations [5] and a possible decrease in survival [6]. However, psychosocial care of cancer patients has often been underestimated as a key aspect in determining the quality of clinical care [7]. Neglected and untreated symptoms of distress cause an additional burden for the healthcare system because patients are more likely to use healthcare services more frequently and for longer as they undergo further chemotherapy lines [8,9,10].

The assessment of the patient’s psychological discomfort and concerns has been increasingly considered as an indicator of quality of life and comprehensiveness of cancer care both at the clinical and organizational level [11,12,13]. Several studies have shown that 30–40% of cancer patients present various symptoms of emotional distress as a result of diagnosis and treatments [14]. These symptoms range from normal feelings of vulnerability to serious existential crises that can cause severe problems such as depression [15,16,17]. For the majority of people, signs and symptoms of upset represent common temporary adaptation problems, while for others, such reactions may persist or worsen [18,19]. Despite this, <10% of distressed patients are referred by oncologists for mental health consultations or other suitable interventions [20].

At an international level, distress is acknowledged as the “sixth vital parameter” to be monitored regularly in clinical practice, on a par with the other vital parameters such as temperature, heart rate, respiration rate, blood pressure and pain [21,22,23]. In oncology, a specific tool, the Distress Thermometer (DT), was developed to assess distress levels and measure emotional suffering and its possible causes (physical, family, interpersonal, spiritual and practical). The DT was first validated by Roth et al. in 1998 for patients with prostate cancer [24]. Further research expanded the use of DT to screen multiple cancer diagnoses [25]. As clinical judgement alone is considered inadequate to identify distress, the DT was also included in National Comprehensive Cancer Network (NCCN) guidelines as a screening tool for the early detection of patients in need of psychosocial support and specific clinical interventions [26].

An evaluation process supported by adequate referrals is the core of a distress screening program [27]. From a perspective of whole-person cancer care, routine evaluations of emotional distress are essential in clinical practice. Healthcare professionals should be aware that the patient’s emotional state is an integral part of health in the healthcare system. They also should be informed about the other problems patients indicate on the Problem List (PL) because distress often develops from non psychological issues. The PL identifies 36 potential sources of distress containing practical, emotional, family, spiritual, and physical problems, and facilitates clinicians in referring patients for tailored interventions [28,29]. The PL enables patients to describe what they are worrying about and what difficulties they are facing [10].

Distress has a multifactorial etiology and if we only consider the DT score, we risk neglecting the patient’s other perceived needs. Furthermore, studies have revealed that most patients who screen positive for DT do not want to be referred for psychological consultation whereas those with a low DT score often hope for some help [30,31].

Distress screening generally highlights psychosocial issues rather than deeper problems patients are facing [28].

An accurate identification of distress through the use of the DT should be followed by adequate support, but there is little evidence that such support is available or structured appropriately. These discrepancies, as evidenced by some studies, indicate the need to combine the capacity of the DT to identify distress with the health professionals team’s ability to manage and adequately address the symptoms that are the source of the distress [10].

If distress screening can pinpoint patients’ unmet needs, less attention is given to referral and uptake of available supportive services. Distress screening is the first step to assess needs but it is also important to deepen the engagement to screening and to identify the reasons for refusal, in order to implement an efficient policy of the administration of the DT [32].

In Italy, unlike other countries, a national structured policy of screening for distress in oncology has not yet been set up. Several Italian studies have assessed distress in cancer patients, confirming a 40–50% prevalence of the condition, but with little data on the types of problems the patients face in their cancer care settings. No information is available on what happens after distress screening in terms of acceptance for referral or further assessment by psycho-oncologists in those who are significantly distressed from a clinical point of view. Given the importance of communication between healthcare professionals and patients, more data are need in the Italian context. In this regard, we previously showed that distressed Italian cancer patients tended to perceive their doctors as both disengaged and unsupportive, highlighting the need to adjust relational and communication style on the basis of the patients’ psychological condition [33].

The aims of the present study were threefold: (i) to detect the acceptability of distress screening in Italian cancer centers; (ii) to examine the prevalence of distress in relation to cancer-related problems; (iii) and to evaluate patient adherence to referral to the psycho-oncology service for a support program or other necessary interventions.

## 2. Results

Of the 406 patients contacted by nursing staff for stress assessment, 112 declined to take part in the study for several reasons, i.e., 61 not interested; 11 thought the study not useful; 10 for personal problems; eight for being too weak or physically impaired; four for lack of time; and 18 for other reasons. The patients who refused were more likely to be older, retired, at a more advanced stage of illness, and with no previous experience of psychological intervention compared to those who accepted (*n* = 294, 72% of the total population approached). Complete data on distress assessment were therefore available for 293 subjects. Table 1 shows the sociodemographic and clinical characteristics of the participating patients.

Using the rule-out scoring system on the DT score, 151 (51.5%) patients had no-low distress (score 0–3), 64 (21.8%) had mild distress (4–5) and 78 (26.6%) had moderate to severe distress (score ≥ 6). There was no difference between the three DT score groups in relation to patient characteristics. Conversely, a substantial difference was seen between patients who had or had not undergone a psychological consultation, with higher DT scores in the latter group (*p* = 0.0006). Significant differences among the three DT groups were also found in the PL areas involving practical, relational, emotional and spiritual issues, but not physical issues (Table 2). Taking into account absolute and percentage values, physical problems affected >80% of patients in the three DT subgroups, whereas other areas showed lower percentages of representativeness.

Of the 78 (26.6%) patients with a score ≥6 who were referred to the Psycho-Oncology Service, 51 (65.3%) accepted the telephone interview, representing a study adherence of 65.4%. Of these, five (6.4%) subsequently changed their minds and refused to participate. Twenty-two (28.2%) patients rejected the initial invitation immediately for a wide range of reasons, i.e., logistic difficulties (*n* = 4), physical problems (*n* = 3), belief that psychological services were not useful (*n* = 3), no interest in emotional issues (*n* = 2), already in the care of psychological healthcare professionals (*n* = 4), feeling good despite the high DT score, too busy, and feeling embarrassed about opening up to a psychologist.

No significant differences were found in the five areas of the PL between those who accepted psychological counselling and those who did not (data not shown). Of the remaining 51 who accepted the interview, 14 (27.5%) had had at least one psychological consultation prior to the study, of whom nine had continued seeing their therapist, two were referred to a Psychological Support Service and three had chosen not to continue along this path. Among the 37 (72%) patients who had never received any psychological support, 11 were referred to a Psychological Support Service and 26 were not clinically deemed to be in need of psychological intervention.

## 3. Materials and Methods

### 3.1. Design and Participants

**Figure 1:**
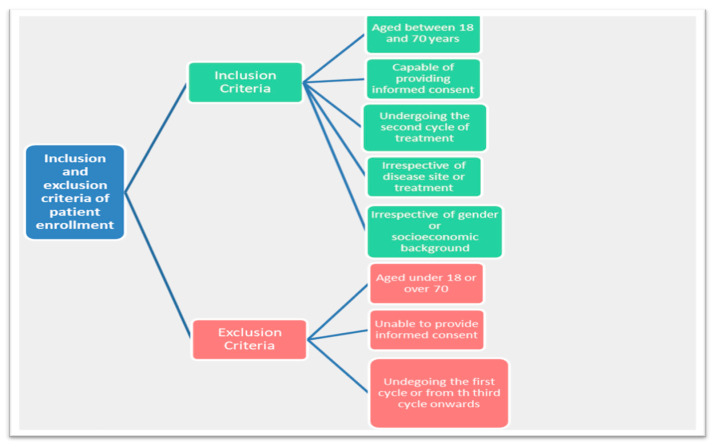
Criteria of inclusion and exclusion.

The study was carried out at the Cancer Outpatient Units of three Italian hospitals in the Emilia-Romagna region of northern Italy, i.e., IRST IRCCS in Meldola, Infermi Hospital in Faenza and University Sant’Anna Hospital in Ferrara. It was approved by Ethics Committee of Romagna (CEROM) and performed in accordance with the ethical standards outlined in the 1964 Declaration of Helsinki. A protocol amendment was made in 2018 to increase study sample size. Participants were cancer outpatients aged between 18 and 70 years, capable of providing informed consent and undergoing the second cycle of treatment with chemotherapy or biological therapy, irrespective of gender, disease site or treatment setting, and from a variety of socioeconomic backgrounds (Figure 1). We chose this treatment setting to avoid first-cycle anxiety and to limit DT bias [16,34].

All patients were informed of the aims of the study and gave written consent to participate.

### 3.2. Measurement

The DT and PL were used to assess distress and patient concerns or problems. The tool, developed by the National Comprehensive Cancer Network (NCCN) [35] and validated in Italy [14,36], consists of two parts: the DT, which is a visual analog scale evaluating the subject’s perceived level of distress in the previous week on a scale from 0 (no distress) to 10 (maximum distress); and the PL, which is a list of 36 problems grouped into 5 categories, i.e., practical, family/relational, emotional, spiritual, and physical. The patient records the presence of such problems in the previous week by ticking “yes” or “no” [26,37]. This simple tool could easily be integrated into a distress management program [10].

According to the Italian validation of the DT, a cut-off score of 5 identifies patients experiencing significant distress [14]. The recent version of NCCN practice guidelines for the management of distress indicates that a DT score of 4 or more has clinical significance [29]. Although it is acknowledged that demographics, language, culture and setting influence distress cut-off scores in research involving mixed samples, the majority of studies agree on a DT cut-off score of 4 or 5 [14,38,39,40,41]. More specifically, a score of 4–5 is considered as mild distress, while scores of >6 are more clinically significant (moderate distress, DT scores of 6 and 7; severe distress, DT scores >8) and deserving of specialist attention because they are associated with psychopathological conditions.

### 3.3. Definition of Measures

We defined the prevalence of medium-high distress as the number of patients with a DT score ≥6 out of the total number of patients who completed the DT. DT acceptability was defined as the number of completed questionnaires out of the total number of returned questionnaires. Study adherence was defined as the number of post-DT interviews conducted out of the number of proposed interviews.

### 3.4. Procedures

The research nurse provided a list of all consecutive eligible patients. Infusion nurses informed patients meeting all inclusion criteria about study procedures, obtained informed consent from those who agreed to take part, and invited them to complete the DT and PL. The nurses involved had undergone training on study procedures and administration of the tools from IRST Psycho-Oncology staff, in accordance with the protocol previously applied in one of the centers participating in this study [42]. Sociodemographic and disease data were taken from patients’ charts and recorded in the case report form (CRF).

We first used a rule-out scoring system to explore the general level of distress among patients indicating no-low distress (distress score: 0–3, 4–5 and >6). Differences in the PL were analyzed for each group. We then used the score 6–7 to identify clinically significant psychological distress in cancer patients [14,18,43].

Patients who reported a DT score ≥6 were referred to the Psycho-Oncology Service and invited to take part in a telephone interview with a psycho-oncologist (PON). The interview was carried out in the same format in all centres, using a specific question checklist. During the phone interview, the availability of patients to undergo a post-DT consultation or their reasons for declining was recorded. Questions asked or requests for clarification made by patients were also registered. The post-DT interview consisted of a clinical assessment of the emotional state of the patient and the problems indicated in the PL.

At the end of the interview, three outcomes were possible; (1) Conversation about the results of the distress assessment; (2) Referral to the Psychology Community Services of the Department of Mental Health or to other physicians (e.g., palliative care clinic); (3) Referral to the Psycho-Oncology Units of a participant’s hospital for a maximum of two consultations designed to assess the patient’s emotional state and level of motivation and to determine whether a structured psychological support is needed.

### 3.5. Data Analysis

Categorical data were expressed as absolute and percentage frequencies, while continuous variables were expressed as median and range. Nonparametric tests were applied to the variables: chi-square or Fisher exact test for categorical variables and Wilcoxon rank sum test for continuous variables. The Cochran-Armitage test for trend was performed to evaluate the association between demographic characteristics and PL items in the three groups defined by the DT score, while the Kruskall-Wallis test was used for continuous variables. *p*-values < 0.05 were considered statistically significant. Statistical analyses were carried out with SAS Statistical software (version 9.3, SAS Institute, Cary, NC, USA).

## 4. Discussion

The present study aimed to explore the practical usefulness of and potential barriers to the application of distress management guidelines in three hospitals. Distress is a multifactorial emotional state, indicating the complexity of its causes. Numerous aspects of a patient’s life contribute to the personal experience of distress, including physical symptoms, disease severity, treatment, level of physical activity and performance status, social support, and psychological factors such as optimism, coping style, and pre-morbid or current depression [44].

Although nurses in the participating centers were trained in screening for distress and in the use of the PL, almost one third of the eligible population did not accept the screening procedure. Our results reflect those of other studies that identified multiple barriers to efficient implementation; the main observed obstacles are lack of willingness, reluctance to discuss emotional issues, perception of stigma, factors such as age or language, time availability, interviewer skills, quality of the relationship between patient and operator, and lack of awareness of available resources [45,46].

Greater efforts are needed to overcome these barriers. For example, information on the availability of a Psycho-Oncology Service could be provided in several ways for example, by disseminating brochures in hospitals or by communicating directly with healthcare providers (including oncologists, cancer care providers and primary care physicians). In addition, an initial online psychological consultation might be helpful for people who live far away from the hospital. The creation of some form of transport service to take patients to and from hospital would be another useful initiative (for this purpose the collaboration with local voluntary and advocacy associations can be extremely useful). Finally, educational programs on the importance of distress screening should be improved by all stakeholders and become an integral part of the cancer healthcare services provided, as already done in other countries (e.g., Canada).

In our study, the fact that the DT was proposed by nurses just before treatment infusion rather than during the visit with the oncologist may have made patients reluctant to disclose concerns and emotional issues. Other factors relating to different cultural and organizational contexts may also play a part in acceptance of distress screening. For example, this service is more widely used in Northern Europe and North America than in Italy.

In order to implement an effective screening for distress further research is needed, in particular it is important to involve multiple levels of cancer care delivery, from patients’ perspectives to healthcare organizations [47].

In our study, around 50% of the patents showed distress scores higher than the cut-off used in the rule-out score systems, and one out of four (26% of the whole sample and 50% of those distressed) patients were clinically significantly distressed (“cases”), requiring intervention. Furthermore, patients who accepted the DT and PL tools were more likely to have received psychological consultation in the past. This underlines that previous positive experiences with psychological disciplines may help patients to acknowledge the need for help in relation to emotional issues. In our study, the majority of patients with clinically significant levels of distress had not received psychological care or did not take advantage of available psychological support resources, refusing the post-DT interview or referral to our Psycho-Oncology Service. This is in agreement with findings from other studies that concluded that a large percentage of distressed patients tend to decline help [48,49]. Refusal has been associated with the sense of stigma related to mental health and psychological services [50], unawareness of available support for distress symptoms, and disinterest in what is traditionally perceived as “talk therapy” [51]. Our results are also in line with those of other studies underlining the importance of communication skills training and referral management guidelines for healthcare operators [52,53]. Among those who did not consent to take part in our study, the number of retired individuals was significantly higher than that those in employment.

Greater attention should be paid to this group so that data on distress and related problem areas can be collected during medical visits or anytime during the course of the doctor-patient relationship. It would also be useful to find alternative ways to recruit these cancer patients. In this respect, training in communication skills and referral management is worthwhile for all healthcare providers and could improve the adherence of retired patients. Furthermore, the involvement of primary care and other medical specialists (both in terms of specialization for the several forms of cancer, and life-span, young, adult, and old age) would help patients to understand the importance of taking part in a distress screening program.

Although studies have shown the effectiveness of psychological intervention in patients with distress [54,55], our findings underline that distress may also have causes of a different nature, such as adverse physical reactions to treatments, for which psychological consultation is not appropriate [10]. We also observed that a small number of “cases” (around 25%) interviewed by telephone were already undergoing some form of psychological treatment, and the other two-thirds were referred to psycho-oncology or psychological services for the first time. However, we cannot know the final outcome of the referral because, as shown in other studies, a percentage of patients tend to drop out [56]. This highlights the reticence of patients to disclose their perceived distress and could be an issue worthy of further exploration in the ongoing discussion on the implementation of distress screening [10].

There are a number of limitations to the study, the most important being the relatively small sample size and involvement of only a few centers. This could have indicated potential differences in different parts of the country in terms of both response to screening and referral. A further limitation is that our study focused on a one-time use of the DT and PL, whereas screening for distress should be a routine part of clinical care and carried out at each visit, especially when diagnoses are made and during clinically meaningful or stressful events that occur during the course of the disease [57,58] From a clinical point of view, this would almost certainly improve patient recruitment, including those who refused the initial screening.

The study also has a number of strengths, e.g., it was carried out in three Italian institutes with a well-established Psycho-Oncology Service, access to which is possible through healthcare providers or through a request from family members or from patients themselves. Our POS operates within the National Health Service and is part of standard clinical practice. Information about the service is provided in various ways, e.g., via brochures or through direct communication with healthcare providers.

In our study, the barriers to the uptake of supportive services and the percentage of patients who refused help highlight the need to further improve the training of healthcare professionals in the area of distress screening, especially in relation to less compliant patients.

## 5. Conclusions

Distress arises from both psychological causes and from other clinical and physical causes, such as adverse reactions to treatments or practical issues. The present Italian study confirms that screening *per sé* is insufficient to deal with the problem of distress and that more emphasis should be placed on implementing referral and treatment. Screening alone can be considered as part of a global, comprehensive person-centered approach to facilitate the detection of distress. However, it has been seen that those who accept screening and show significant clinical levels of distress requiring specialist support (psycho-oncology) tend to refuse help. Thus, irrespective of the implementation of a distress screening policy at institutional level, more should be done to reach the highest possible number of patients and to motivate the highest possible number of patients to be referred to Psycho-Oncology Services when needed. Healthcare professionals approaching patients must attempt to destigmatize the concept of distress screening, comprehensively explore all possible aspects contributing to distress. They should also focus on the motivational aspects of patients’ disclosure, in order to tailor a suitable referral. A multidisciplinary approach is worthwhile to increase the benefit of screening of distress and to improve uptake.

## Figures and Tables

**Table 1:** Patient characteristics.

Characteristics	No DT Questionnaire	DT Questionnaire	Total	*p*-Value ^#^
Total no. patients	112	294	406	-
Median age, years (range)	63 (36–73)	59.5 (21–79)	60.5 (21–79)	0.0174
**Gender**				
Male	43 (38.4)	113 (38.4)	156	0.9937
Female	69 (61.6)	181 (61.6)	250	
**Children (*n*)**	11 (15.9)	42 (17.5)	53	0.5858
None	19 (27.5)	79 (32.9)	98	
One	39 (56.5)	119 (49.6)	158	
Two or more	43	54	97	
**Occupation**				
Worker	26 (36.6)	124 (45.1)	150	0.0080
Retired	37 (52.1)	91 (33.1)	128	
Other (housewife, unemployed, student)	8 (11.3)	60 (21.8)	68	
Unknown	41	19	60	
**Primary site of disease**				
Breast	29 (25.9)	95 (32.3)	124	0.3227
Gastrointestinal tract	24 (21.4)	70 (23.8)	94	
Lung	13 (11.6)	29 (9.9)	42	
Urogenital tract	12 (10.7)	40 (13.6)	52	
Hematologic malignancy	14 (12.5)	28 (9.5)	42	
Other location	20 (17.9)	32 (10.9)	52	
**Treatment setting**	79 (76.0)	179 (63.7)	258	0.0231
Advanced	25 (24.0)	102 (36.3)	127	
Neoadjuvant/adjuvant	8	13	21	
**Previous psychological contact**				
No	107 (96.4)	249 (85.6)	356	0.0014
Yes	4 (3.6)	42 (14.4)	46	
Unknown	1	3		

DT, digital thermometer. ^#^ *p*-value from chi square test or Fisher exact test as appropriate.

**Table 2:** Analysis of the PL of single subgroups for DT score.

PL	DT 0–3 (*n* = 138)	DT 4–5 (*n* = 56)	DT ≥ 6 (*n* = 68)	Total (*n* = 262)	*p*-Value ^#^
**Practical problems**					
No.(%) patients with ≥1 practical problem	22 (15.9)	21 (37.5)	33 (48.5)	76 (29.0)	<0.0001
Median no. practical problems (range)	1 (1–2)	1 (1–3)	1 (1–4)	1 (1–4)	-
**Relational problems**					
No.(%) patients with ≥1 relationship problem	12 (8.7)	9 (16.1)	23 (33.8)	44 (16.8)	<0.0001
Median no. relationship problems (range)	1 (1–2)	1 (1–2)	1 (1–3)	1 (1–3)	-
**Emotional problems**					
No.(%) patients with ≥1 emotional problem	82 (59.4)	48 (85.7)	62 (91.2)	192 (73.3)	<0.0001
Median no. emotional problems (range)	2 (1–6)	3 (1–6)	4 (1–6)	2 (1–6)	-
**Spiritual aspects**					
No.(%) patients with spiritual problems	2 (1.4)	1 (1.8)	10 (14.7)	13 (5.0)	0.0001
**Physical problems**					
No.(%) of patients with ≥1 physical problem	122 (88.4)	49 (87.5)	64 (94.1)	235 (89.7)	0.2500
Median no. of physical problems (range)	3 (1–12)	6 (1–15)	7 (1–15)	5 (1–15)	-

DT, digital thermometer. ^#^ *p*-value from Cochran-Armitage trend.

## Data Availability

The data presented in this study are available upon reasonable request from the corresponding author.
